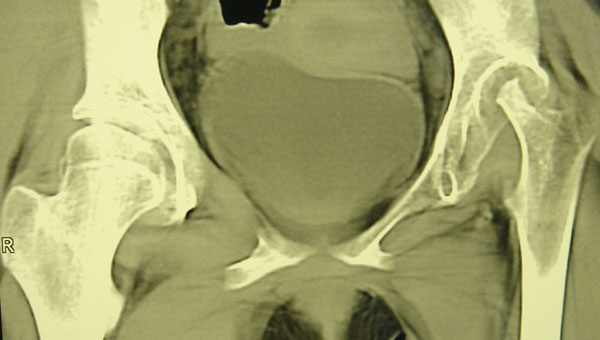# An experience of sequental use of three biologics and recombinant growth hormone in a patient with juvenile idiopathic arthritis associated with unsuccessful outcome of hip damage

**DOI:** 10.1186/1546-0096-9-S1-P143

**Published:** 2011-09-14

**Authors:** IP Nikishina, SR Rodionovskaya, AN Shapovalenko, LY Filippova, OM Kostareva

**Affiliations:** 1 Scientific Research Institute of Rheumatology of RAMS, Pediatric Department, Moscow, Russian Federation; 2 Children`s Hospital №38 Federal Medical Biological Agency of Russia, Moscow, Russian Federation

## Background

Short stature and hip lesions are typical complications of juvenile idiopathic arthritis (JIA) especially in systemic and polyarticular variants with early onset. Recovery of linear growth is quite often reached as result of biologics therapy, but use of recombinant growth hormone (rGH) sometimes is required.

## Case report

A 14-year-old girl with severe JIA is under our observation. Polyarthritis involving the hips joints was formed in second year of her life. Despite for 8 years of continuous treatment by DMARDs (methotrexate, cyclosporin A, leflunomide sequentially), NSAIDs, low doses steroids (<0.3 mg/kg), i.a.injections arthritis was persistent and destructive lesions of left hip was developed. Due to growth retardation rGH treatment 0.05 mg/kg was used with good results for linear growth, but arthritis has worsened. Infliximab was started on 10th year of disease with good efficacy after 3 infusions (50%ACR pedi response). It was discontinued because of severe infusion reactions. Further adalimumab was applied within 7 months with achievement of 30% ACR pedi response by the first 3 months and gradual full loss of efficacy. As a third lines of biologics last 14 months abatacept therapy 10 mg/kg combined in a low doses steroids <0.1 mg/kg and methotrexate 15 mg/m2/week is applied. Efficacy of therapy was achieved 30% -50% -70% -30% ACR pedi response by 3-6-9-12 months. Loss of efficacy is probably connected with the resumption of rGH treatment after 9 months of abatacept use. Dramatic damage in the left hip with total lysis of femoral head was occurred on MRI and CT hip scans in parallel increase of disease activity 3 months later after renewal rGH.

## Conclusion

It seems that action of rGH is referred opposite to action of biologics applied in JIA and may stimulate the progression of osteonecrosis of femoral head.

**Figure 1:**
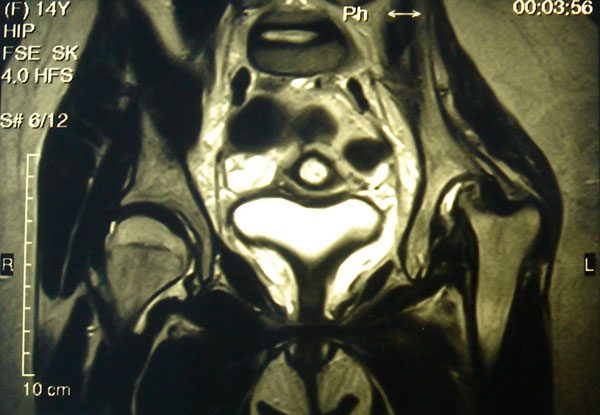


**Figure 2:**